# Whole genome resequencing reveals an association of *ABCC4* variants with preaxial polydactyly in pigs

**DOI:** 10.1186/s12864-020-6690-1

**Published:** 2020-03-30

**Authors:** Cheng Ma, Saber Khederzadeh, Adeniyi C. Adeola, Xu-Man Han, Hai-Bing Xie, Ya-Ping Zhang

**Affiliations:** 1 State Key Laboratory of Genetic Resources and Evolution, Yunnan Laboratory of Molecular Biology of Domestic Animals, Kunming Institute of Zoology, Chinese Academy of Sciences, Kunming, China; 2 Kunming College of Life Science, University of Chinese Academy of Sciences, Kunming, China; 3 University of Chinese Academy of Sciences, Beijing, China

**Keywords:** Preaxial polydactyly, *ABCC4*, Ciliogenesis, Limb development, Whole-genome sequencing

## Abstract

**Background:**

Polydactyly is one of the most common congenital limb dysplasia in many animal species. Although preaxial polydactyly (PPD) has been comprehensively studied in humans as a common abnormality, the genetic variations in other animal species have not been fully understood. Herein, we focused on the pig, as an even-toed ungulate mammal model with its unique advantages in medical and genetic researches, two PPD families consisting of four affected and 20 normal individuals were sequenced.

**Results:**

Our results showed that the PPD in the sampled pigs were not related to previously reported variants. A strong association was identified at *ABCC4* and it encodes a transmembrane protein involved in ciliogenesis. We found that the affected and normal individuals were highly differentiated at *ABCC4*, and all the PPD individuals shared long haplotype stretches as compared with the unaffected individuals. A highly differentiated missense mutation (I85T) in *ABCC4* was observed at a residue from a transmembrane domain highly conserved among a variety of organisms.

**Conclusions:**

This study reports *ABCC4* as a new candidate gene and identifies a missense mutation for PPD in pigs. Our results illustrate a putative role of ciliogenesis process in PPD, coinciding with an earlier observation of ciliogenesis abnormality resulting in pseudo-thumb development in pandas. These results expand our knowledge on the genetic variations underlying PPD in animals.

## Background

Polydactyly is one of the most commonly observed congenital limb malformations and ciliopathies. This abnormality is characterized with additional digits in fingers or toes and has been reported to be in association with dozens of genes and complicated diseases [1]. Polydactyly constitutes the highest proportion among the congenital limb defects in various epidemiological surveys, but its regulation mechanism has not been well understood [2]. Based on the anatomic position of the additional digits, polydactyly can be classified into preaxial polydactyly (PPD), postaxial polydactyly and central polydactyly [3]. Previous pathological researches have shown that most of the PPD abnormal cases follow an autosomal dominant inheritance pattern and a few express an autosomal recessive pattern of inheritance [4]. In addition to high incidence in humans, there is high morbidity rate in pigs, cats, chickens and other vertebrates [5].

Previous studies have shown that the vast majority of polydactylies are associated with Sonic Hedgehog (SHH) signaling pathway and ciliogenesis process [6]. The SHH signaling is an evolutionary highly conserved signal transduction pathway that plays critical role in specifying the growth and polarity of vertebrate limb buds. In the process of limb development, the Zone of Polarizing Activity (ZPA) signaling center determines the formation of anterior-posterior axis of limb buds [7]. The ectopic expression of *SHH* gene in the anterior part of limb bud is the main causative agent of PPD. Normally, SHH signal is only expressed at the posterior portion of ZPA region of limb bud [8, 9]. The disruption or mis-regulation of SHH pathway often results in congenital birth defects, such as holoprosencephaly and polydactyly [10]. Lettice et al. [11–14] have found that the disruption of Zone of Polarizing Activity Regulatory Sequence (ZRS), a long-range cis-regulator for *SHH* located in the fifth intron of *LMBR1* gene, results in the ectopic expression of *SHH* responsible for PPD. Many single nucleotide polymorphisms (SNPs) in ZRS have been reported to be in association with PPD [15].

In the early stages of embryonic development, *Gli3* polarizes the limb into anterior-posterior axis through the antagonism with *HAND2* [16, 17]. *SHH* mediates the limb patterning by regulating *Gli2* and *Gli3*, which act as full-length transcriptional activators (GliA) in the presence of *SHH* and are cleaved into a short form as a truncated repressor (GliR) in its absence [18, 19]. *Gli3* mutant limbs are characterized by severe polydactyly and associated with ectopic anterior expression of *Hoxd* gene [20, 21].

Additionally, as an important signal transduction and sensory center in eukaryotic cells, cilia play a crucial role in SHH signal transduction and regulation of its downstream target genes [22–24], such as *Gli* gene family [25]. The disruption or mis-regulation of ciliogenesis process results in serious ciliopathies, including polydactyly [23, 26]. Abnormalities in cilia due to the defects of *DYNC2H1* in retrograde intraflagellar transport (IFT) have caused the short-rib polydactyly syndrome in human [27]. Moreover, defects of DYNC2H1 and PCNT proteins in IFT and ciliogenesis process can cause pseudo-thumb (PPD) both in red and giant pandas [28]. *CYLD* (cylindromatosis) mediates ciliogenesis by deubiquitinating Cep70 and inactivating *HDAC6*, and *CYLD* knockout mice exhibit polydactyly [29]. In addition, previously reported candidate genes associated with polydactyly traits include: *EN2* [30], *MIPOL1* [31], *TWIST1* [32], *PITX1* [33] etc. and reviewed in Malik et al. [2] and Deng et al. [4]. Among all of these identified candidate genes, majority of them are involved in the SHH signaling pathway or ciliogenesis process.

In this study, four pigs from two pedigrees of Large White pig breed were identified with PPD deformity. We aimed at screening for the genomic variants of PPD phenotype through whole genome association studies based on high quality resequencing data. Genetic differentiations between PPD affected and normal groups were calculated and identification of ATP Binding Cassette Subfamily C Member 4 (*ABCC4*) (NCBI gene access ID: 100152536) strong association with PPD phenotype. Furthermore, a missense mutation in *ABCC4* was detected but not in large normal samples. These findings highly prompt us in hypothesizing that *ABCC4* is probably a new candidate gene for PPD through the regulation of ciliogenesis.

## Results

### Pedigree and phenotypic analysis

**Fig. 1:**
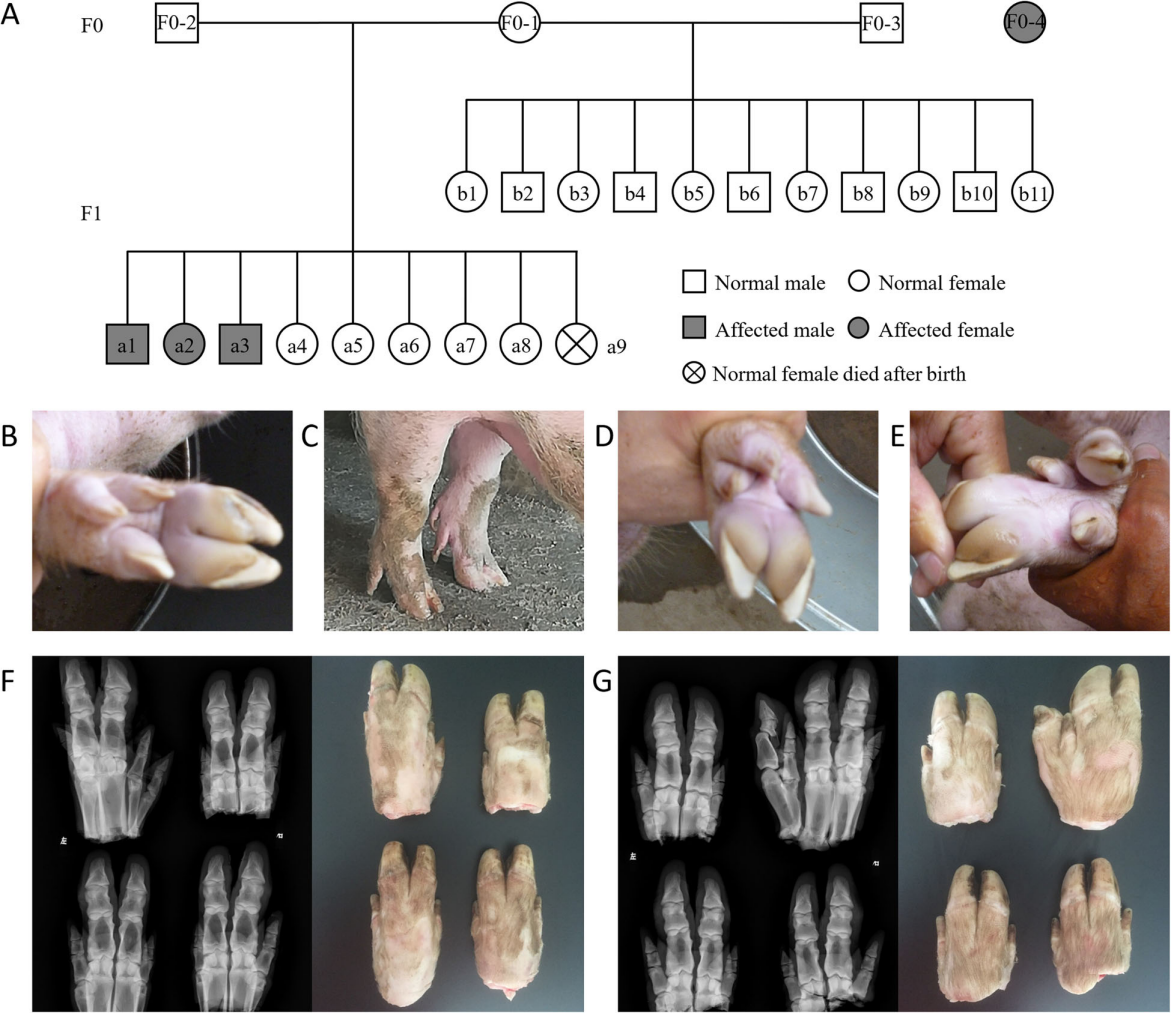
Pedigree and phenotypes information of the four PPD affected pigs. **a** Pedigree of the three PPD affected pigs and one random case. Squares represent males and circles represent females. Shaded symbols denote polydactyly pigs. **b** Phenotypes of the affected male (F1-a1) which expressing a preaxial polydactyly on left forelimb. **c** Affected female (F1-a2) expressing a preaxial polydactyly phenotype on right side of fore limb. **d** Affected male (F1-a3) appears a preaxial polydactyly phenotype on right side of fore limb. **e** Affected female (F0–4) appears a preaxial polydactyly phenotype on left forelimb. **f** Radiograph and phenotype of the affected male (F1-a1). **g** Radiograph and phenotype of the affected male (F1-a3). The four hooves were placed based on the position of alive pigs, at the front were forelimbs and on the right were right limbs

In this study, four PPD affected individuals were collected from two separate families and their genealogical information are shown in Fig. 1a. All of the four PPD pigs were affected at one side of the forelimb, the F1-a1 male (Fig. 1b) and the F0–4 female (Fig. 1c) were affected on the left side of the forelimb, the F1-a2 female (Fig. 1d) and F1-a3 male (Fig. 1e) were affected on the right side of the forelimb.

To show the detail of the additional digit and for further classification, two affected pigs (F1-a1 and F1-a3) were euthanized to get the hooves for radiograph analysis. The detailed phenotypes and radiograph information of F1-a1 and F1-a3 are shown in Fig. 1f and Fig. 1g, respectively. According to the classification method proposed by Wassel [3], the PPD phenotypes in this study are classified into PPD type VI (duplicated metacarpal). Except for an extra toe on anterior side of the forelimb, there were no additional abnormal phenotypes observed in appearance and behavior.

### Characterization of variants

To improve the reliability of data quality and called variants, the genome was sequenced with a higher depth varying from 29.06× (F1-b9) to 38.34× (F0–4) and on average 34.10×, and the average coverage with respect to the pig reference genome sequence (*Sus scrofa* 10.2) is 88.79% (Additional file 1: Table S1). After applying stringent quality control criteria, we identified a total of 13,624,224 SNPs and 2,903,785 Insertion/Deletion (INDELs) in the whole genome and most of them were located in noncoding sequences (Additional file 2: Table S2).

### Reported candidate genes analysis

To investigate whether this PPD phenotype was caused by candidate genes mutations previously identified in human or other species. We scanned the homologue genes of all these candidate regions, but there were no mutations in the protein coding regions, untranslated regions (UTR) sequences, transcription factor binding sites and other highly conserved sequences surrounding the PPD affected individual’s candidate genes. Variants in intergenic region (IGR), intron and other regions were not significantly differentiated between PPD affected and normal pigs, so we ruled out the possibility of the previously reported candidate genes as the causing loci for this PPD phenotype in our study.

### Screening of variants associated with PPD phenotype

**Fig. 2:**
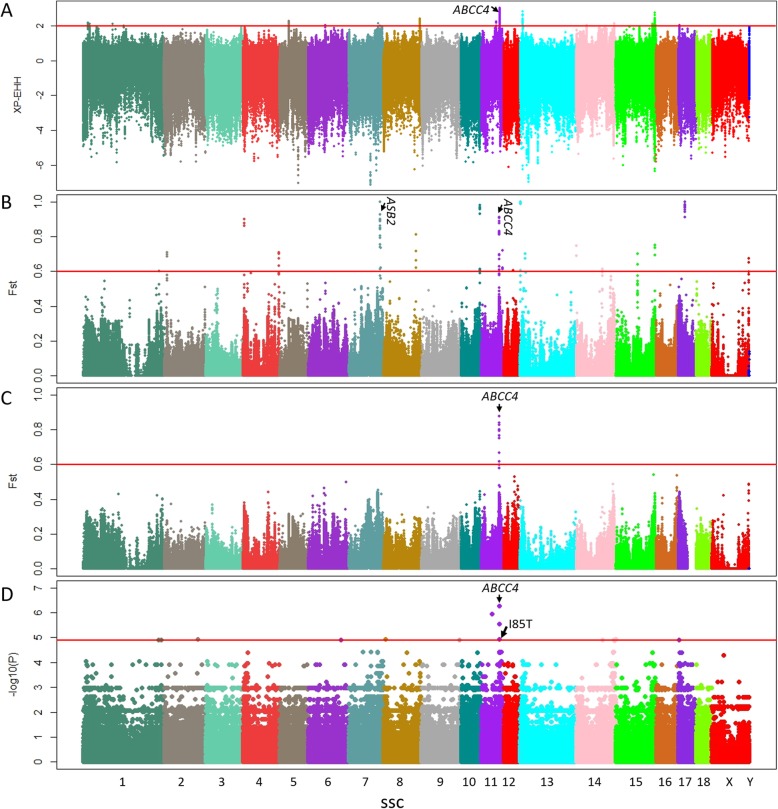
Manhattan plots show the whole-genome screening of putatively loci which associated with PPD. **a** Genome wide SNPs plot of XP-EHH between the affected and normal individuals. **b** F_ST_ plot between the affected and normal individuals based on whole-genome SNPs. **c** F_ST_ plot between the affected and normal individuals based on whole-genome INDELs. **d** Whole-genome association analysis of PPD phenotype

For screening of some haplotypes which were inherited by the PPD affected individuals (but not exist in unaffected groups). We calculated the Cross Population Extended Haplotype Homozygosity (XP-EHH) value [34] between PPD affected and unaffected groups based on whole-genome SNPs. Our results showed that a haplotype including *ABCC4* gene on SSC11 (*Sus scrofa* chromosome 11) was highly associated with PPD phenotype (Fig. 2a). We further located and annotated the highly remarkable regions with mean XP-EHH value larger than 2 (XP-EHH > 2) (Additional file 3: Table S3). Among these regions, SNPs in *ABCC4* region had the most significant value and counted for the biggest proportion of 70.49% in all the highly remarkable SNPs.

Moreover, the fixation index (F_ST_) [35] analyses results showed that the region of *ABCC4* gene on SSC11 was highly differentiated between the affected and normal groups based on both SNPs (Fig. 2b) and INDELs (Fig. 2c). All highly differentiated windows (Weighted F_ST_ > 0.6 and number of SNPs≥10 in each slid window) based on whole-gnome SNPs (Fig. 2b) are listed in Additional file 4: Table S4. All of these regions were located in IGR except *ABS2* on SSC7 and *ABCC4* on SSC11 (Fig. 2b). In addition, we checked these highly differentiated signal regions and further analysis showed that the genotypes did not fully correspond with phenotypes of these regions, and there is no literature showing that these regions are associated with polydactyly. We combined results from haplotype stretches (XP-EHH), SNP differentiation, INDEL differentiation and association analysis to identify the *ABCC4* as a candidate gene. For some other regions, there were some signatures in SNP differentiation, but they were not replicated in other paralleling analysis. Within these highly differentiated window regions, SNPs with F_ST_ > 0.6 were annotated and listed in detail (Additional file 5: Table S5). Among these highly differentiated SNPs, 61.65% of them were located in intergenic regions, 20.62% in introns and only one missense mutation (NC_010453.4:g.70439379A > G) in the third exon of *ABCC4* (Additional file 6: Figure S1). Meanwhile, the highly differentiated regions (Weighted F_ST_ > 0.6 and number of INDELs≥10 in each slid window) of INDELs (Fig. 2c) are listed in Additional file 7: Table S6. All of these regions were located in *ABCC4* gene.

Furthermore, whole genome association study was performed to further analyze the effective SNPs of this deformity based on the basic case-control association test model of PLINK [36] (Fig. 2d). We also identified some highly associated SNPs which were located in *ABCC4* on SSC11, including the missense mutation (NC_010453.4:g.70439379A > G, *P* = 1.19 × 10^− 5^). The top ten highly associated SNPs (*P* ≥ 1.19 × 10^− 5^) are listed in Table 1 and eight of them were also included in the list of highly differentiated SNPs identified through genome scanning (Additional file 5: Table S5).

**Table 1:** Genome association analysis and genotyping results of the top 10 SNPs

SNP ID	Pos (*Ssc* 10.2)	Region	Ref	Alt	GT_A (4)	GT_U (13)	CHISQ	*P* value
*rs791053563	***11:70808545***	***Intron***	***G***	***C***	***0/0 (4)***	***1/1 (11), 0/1(2)***	***25.11***	***5.422E-07***
rs709805150	11:42738578	IGR	C	T	1/1(3), 0/0(1)	0/0(13)	23.68	1.138E-06
*rs711914258	***11:71068779***	***Intron***	***T***	***C***	***1/1(4)***	***0/0(11), 0/1(1), 1/1(1)***	***21.87***	***2.911E-06***
*rs342954583	***11:71068862***	***Intron***	***C***	***G***	***1/1(4)***	***0/0(11), 0/1(1), 1/1(1)***	***21.87***	***2.911E-06***
*rs336503862	***11:71068919***	***Intron***	***C***	***G***	***1/1(4)***	***0/0(11), 0/1(1), 1/1(1)***	***21.87***	***2.911E-06***
rs320384943	11:70801220	Intron	T	C	1/1(4)	0/0(11), 1/1(2)	19.18	1.19E-05
*11:70439379	***11:70439379***	***Exon***	***A***	***G***	***1/1(4)***	***0/0(9), 0/1(4)***	***19.18***	***1.19E-05***
*11:71068754	***11:71068754***	***Intron***	***A***	***G***	***1/1(4)***	***0/0(10), 0/1(2), 1/1(1)***	***19.18***	***1.19E-05***
*rs335010523	***11:71068848***	***Intron***	***T***	***A***	***1/1(4)***	***0/0(10), 0/1(2), 1/1(1)***	***19.18***	***1.19E-05***
*rs325300849	***11:71068895***	***Intron***	***A***	***G***	***1/1(4)***	***0/0(10), 0/1(2), 1/1(1)***	***19.18***	***1.19E-05***

*Pos* Physical position, *Ssc* Sus scrofa, *Ref* Reference allele, *Alt* Altered allele, *GT_A* Genotypes of cases, *GT_U* Genotypes of controls; The number “0” represent reference allele and “1” represent altered allele; The number behind the genotype in brackets represent the number of this genotype’s individuals; CHISQ: Basic allelic test chi-square (1df); P: Asymptotic *p*-value for this test; *IGR* Intergenic region. The bold italic highlighted rows (*) represent the SNPs which identified by genome scanning (Additional file 5: Table S5)

To exclude the possibility of PPD in two families with different genetic causes, the affected individual F0–4 was removed in all repeated analyses and similar results were obtained (Additional file 8: Figure S2). These results highly indicate the possibility of *ABCC4* gene as a new candidate gene for PPD abnormal. Moreover, previous findings revealed that *Iguana/DZIP1* (DAZ Interacting Zinc Finger Protein 1) is an important protein coding gene located in around 800Kb upstream of *ABCC4* (Additional file 9: Figure S3), and was also related to the Hedgehog signaling pathways [37, 38] with an important role in cilium formation [39]. But in this study, we did not detect any variant of significant differentiation between PPD affected and normal groups in *DZIP1*. From these evidences, we concluded that *ABCC4* gene on SSC11 is probably associated with an important role in the early limb formation stage and may affect the formation of PPD.

We further conducted copy number variation (CNV) and long-range structural variants (SV) analysis to explore putative association between PPD and CNVs/SVs. The genome coverage, sequencing depth and mapping quality at the identified putative loci were analyzed in all individuals using the Integrative Genomics Viewer (IGV) [40]. The result showed that no large INDELs, CNVs and SVs were located around these candidate regions.

### Variants analysis

**Fig. 3:**
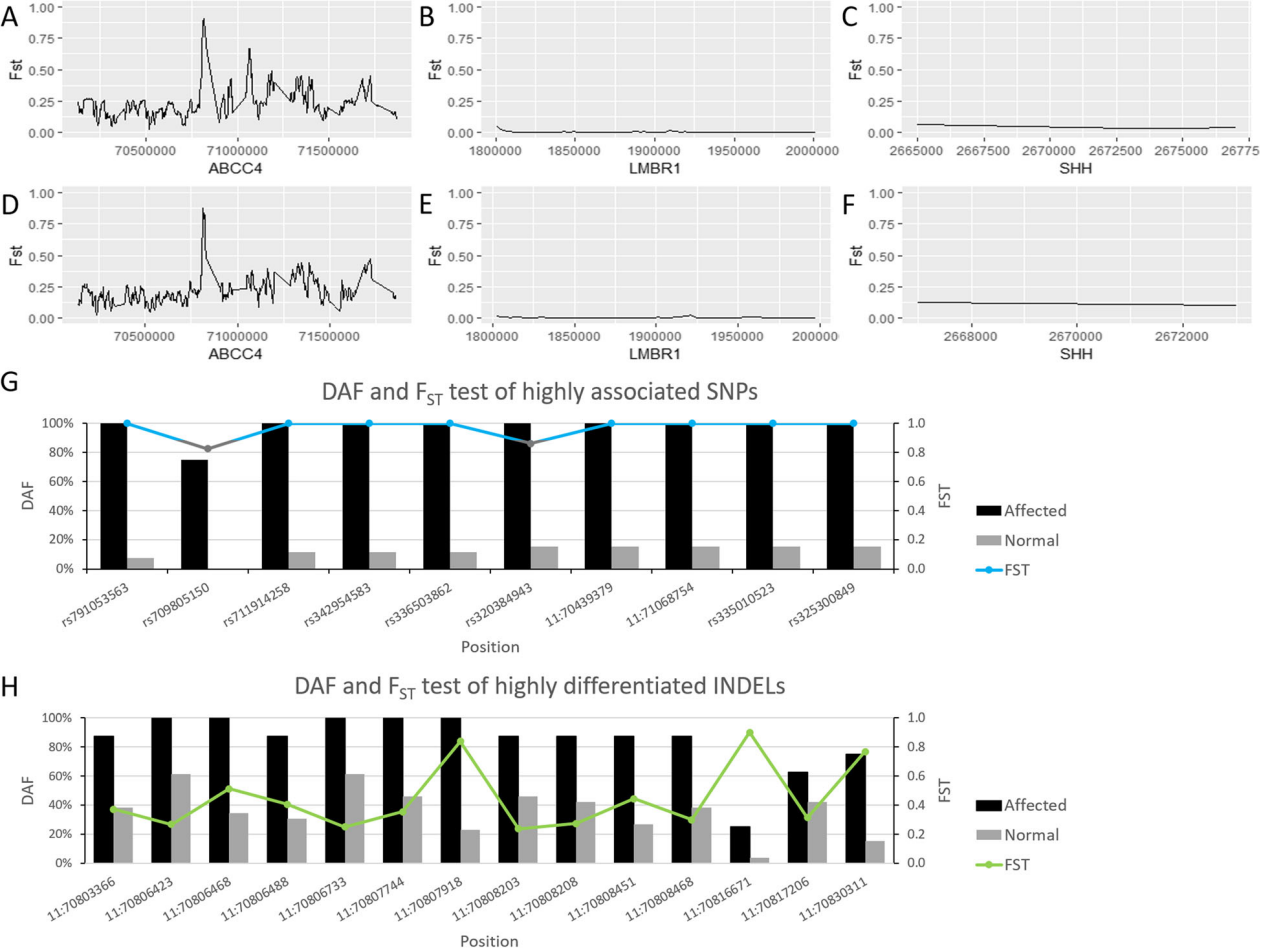
Comparison of candidate genes and DAF analysis of candidate variations. **a**-**c** F_ST_ plot based on SNPs of three candidate genes. **d**-**f** F_ST_ plot based on INDELs of these genes. **g** DAF analysis of the top ten highly associated SNPs. **h** DAF analysis of 14 highly differentiated INDELs around *ABCC4*

In order to further compare the genetic differentiation between PPD affected and normal groups in the two major candidate genes (*SHH* and *LMBR1* (ZRS region was covered)) and *ABCC4*, pairwise F_ST_ analysis was carried out based on SNPs and INDELs (Fig. 3a-f and Additional file 10: Figure S4). The results showed that the genetic differentiation in *ABCC4* region was significantly higher than *LMBR1* and *SHH* between affected and normal groups, and there was no significant differentiation in *LMBR1* and *SHH* gene between the two groups (Fig. 3a-f). The results of SNPs (Fig. 3a-c) and INDELs (Fig. 3d-f) were consistent. In addition, the density of variants in *ABCC4* was higher than *LMBR1* and *SHH* (Additional file 10: Figure S4).

For further identification of the causing mutation of PPD, the top ten highly associated SNPs identified through whole genome association study (Table 1) were selected to calculate the derived allele frequency (DAF) (Fig. 3g). The results showed that these SNPs had significant difference between PPD affected and normal groups, which were in concordance with the results obtained in whole genome screening. In addition, phased genotyping results showed that all of these SNPs were homozygous mutations in all affected individuals except the most significant SNP rs791053563 and F1-a3 in rs709805150 (0/0) (Table 1 and Additional file 11: Table S7).

Furthermore, to investigate the genotypes of the highly differentiated INDELs between affected and normal pigs, 14 INDELs from the highly differentiated window regions (Additional file 7: Table S6) with which F_ST_ > 0.2 were selected for further genotyping (Additional file 12: Table S8). Among these highly differentiated INDELs, eight of them were insertions and six were deletions, all of these were located in intron and far from the nearest exon. The results showed that there was a high allele frequency of mutant in this population and most of which were homozygous mutations. The derived allele frequency of these INDELs showed that there was a remarkable difference between PPD affected and normal groups (Fig. 3h). However, the genotypes of these INDELs were not in well concordance with the phenotype (Additional file 12: Table S8). So, we are not certain whether these INDELs contribute to the mutation in PPD. We detected the noticeable signal of these INDELs possibly due to the linkage disequilibrium.

### Analysis of *ABCC4* mutations

**Fig. 4:**
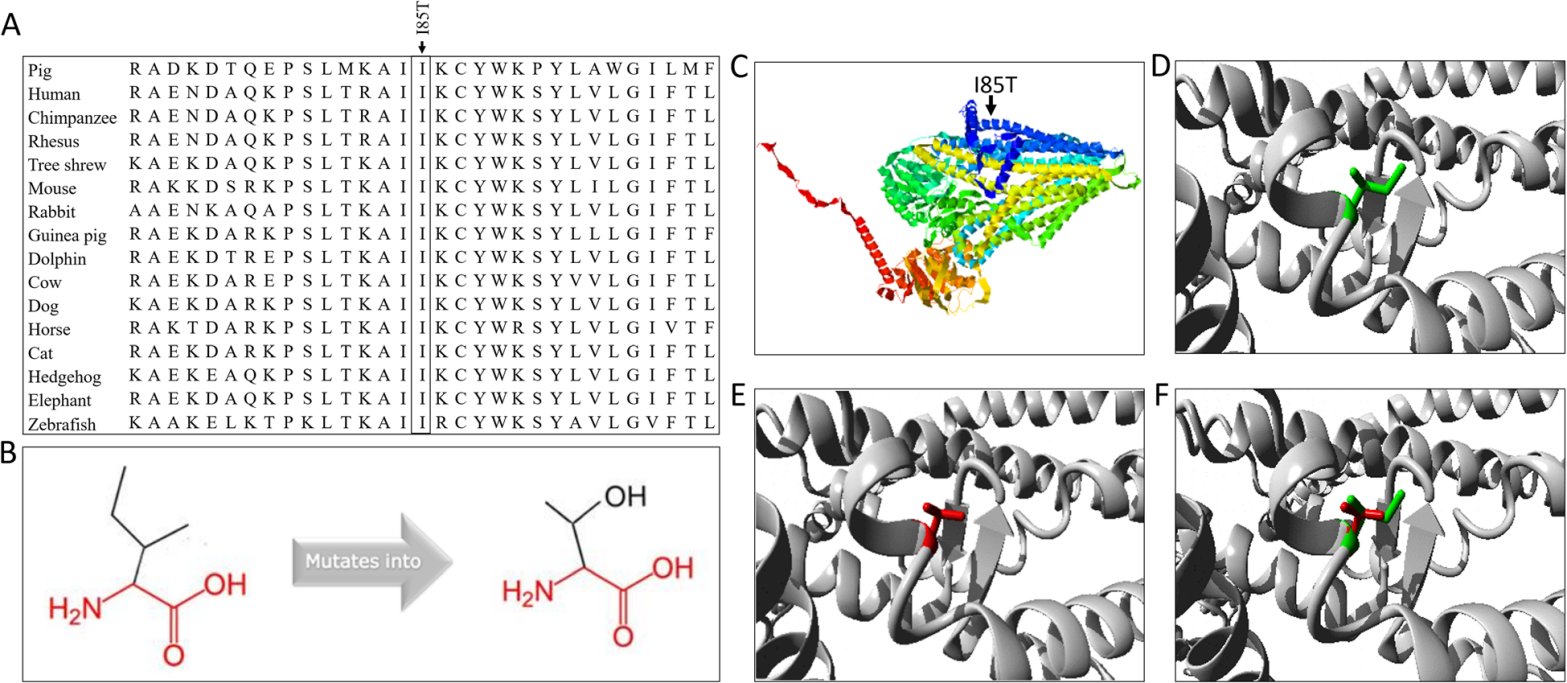
Conservation analysis and structure prediction of the missense mutation site. **a** Cross-species alignment of amino acids sequences of the SNP in *ABCC4*. **b** Changes in the primary structure of this amino acid residues. **c** The full 3D structure of the protein encoded by *ABCC4*. **d** The 3D structure of isoleucine residues domain (wild-type). **e** The 3D structure of threonine residues domain (mutant). **f** Merged structure of wild-type (green) and mutant (red)

In order to further investigate the potential effect of highly associated mutations in *ABCC4* on PPD abnormal. We focused on the missense mutation (NC_010453.4:g.70439379A > G) on the third exon of *ABCC4*, which resulting in the change of isoleucine to threonine (I85T). Firstly, we captured the 60 bp sequence around this SNP and aligned it to the *Sus scrofa* 10.2 and *Sus scrofa* 11.1 reference genomes to further ascertain the position of this missense mutation and eliminate the mapping error. The results showed that the identity of sequence alignment between 11:70439350–70,439,409 of *Sus scrofa* 10.2 and 11:64267163–64,267,222 of *Sus scrofa* 11.1 is up to 100% (Table 2), both regions were annotated at the third exon of *ABCC4* and the variant ID of this SNP is rs1110129849 on *Sus scrofa* 11.1. Cross-species alignment of this protein region showed that this locus was highly conserved in vertebrates (Fig. 4a), indicating that this locus likely has an important biological function under strong evolutionary constraint.

**Table 2:**
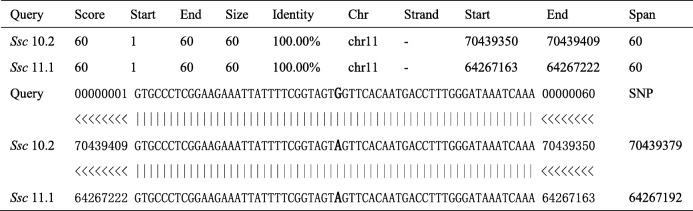
Blast results of different reference genomes

The bold and enlarged positions represent the missense mutation SNP (NC_010453.4:g.70439379A > G)

**Fig. 5:**
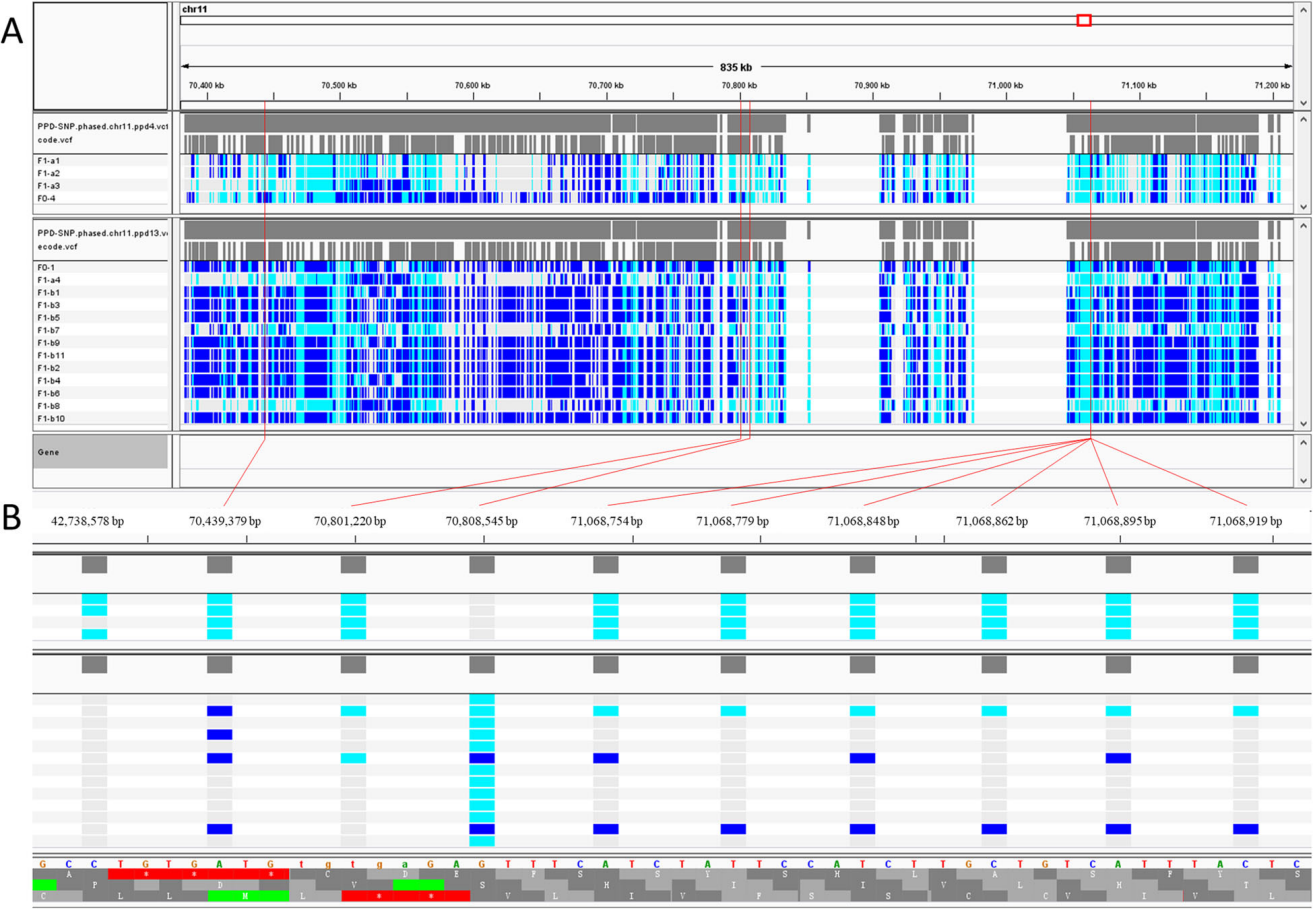
Haplotype pattern around ABCC4 and the genotype of the top ten highly associated and significant SNPs. **a** Haplotype pattern comparation between PPD affected and normal groups in ABCC4. **b** The genotype of the top ten highly associated and significant SNPs

Moreover, our result showed that the allele frequency of this mutation is 0.3529 in the two families, but we didn’t detect this homozygous mutation (G/G) in the large unpublished samples of normal individuals (559 individuals covering 66 domestic pig breeds and wild boars) from our lab (Additional file 13: Table S9). Only seven of them were heterozygous (A/G) (Additional file 14: Table S10) but four of them were filtered by quality control. The statistical results of the derived allele frequency showed that there was a great difference between population in this study and unpublished data. The potential association between this nonsynonymous mutation and PPD phenotype by genotyping illustrated that all of the four affected pigs were mutant homozygous (G/G). There were two kinds of genotypes in normal individuals, nine of them were wild type homozygous (A/A) and four individuals were heterozygous (A/G) (Table 1 and Additional file 11: Table S7). In addition, the most significant SNP (rs791053563, *P* = 5.422 × 10^− 7^) identified by association study was located in the intron region, and interestingly, all the affected individuals were homozygous wild-type at this locus, while the normal individuals were mutant genotypes at this locus. Besides, we have verified this SNP in the large normal population (*n* = 559; 66 breeds; 441 no-missing) and found that the majority (82.97%) of these no-missing normal individuals were homologous for ancestral allele (0/0), and some of them were heterozygous (0/1; 9.73%) or homozygous (1/1; 7.30%) for the derived allele (Additional file 14: Table S10). So, the possibility of this SNP as a potential pathogenic mutation was excluded, and other variants were in similar situation, either the individuals with the homozygous mutation were not affected, or the individuals affected were not all homozygous mutation. The haplotype patterns of *ABCC4* region between PPD affected and normal groups are shown in Fig. 5a and all of the ten highly associated and significant SNPs are shown in Fig. 5b.

Furthermore, we used I-Mutant2.0 [41] to predict the protein stability changes for this mutation (NC_010453.4:g.70439379A > G), and the results showed that the protein stability is “decrease”. In addition, we predicted whether this amino acid substitution has an impact on the biological function of the protein encoded by *ABCC4* through PROVEAN [42]. Results showed that the mutation of isoleucine to threonine at position 85 is “deleterious” with the PROVEAN score of − 3.084. The HOPE [43] web site reported that the mutated residue is located in a domain that is important for binding of other molecules, the mutant residue is more hydrophilic than the wild-type residue and might disturb this function. Furthermore, to compare the structural changes before and after mutation of NC_010453.4:g.70439379A > G, we constructed the 3D structure of this protein encoded by *ABCC4* with HOPE [43] and STRUM [44] web server (Fig. 4b-f). The results showed that the mutated residue is located in the alpha helices and is smaller than the wild-type residue. Changes in this amino acid residue might have probably affected the protein’s ability to bind to the membrane or other molecules.

## Discussion

In this research, we utilized pig as animal model in studying polydactyly and identified *ABCC4* as a new candidate gene for PPD abnormal possibly through the regulation of ciliogenesis. A missense mutation was detected in *ABCC4* which might have disrupted ciliogenesis. Our results further confirmed that the pseudo-thumb development in pandas by disrupting ciliogenesis and pointed out the fundamental role of ciliogenesis underlying PPD in different species.

Animal models are crucial in understanding both genetic and non-genetic diseases. Our study focused on pig based on its unique advantages of pig in medical and genetic researches, including more offspring in shorter generation interval, the anatomical similarities to humans (body size, cardiovascular system), functional similarities (gastrointestinal system, immune system) and the availability of disease models (diabetes, atherosclerosis) [45]. Pig has been considered as an ideal animal model to study human diseases.

SHH signal of anterior-posterior axial plays an important role in the early limb development and patterning. The downstream transduction of SHH signal is received and transported by cilia through a complex network [46]. As an important signal center and sensory organelle, cilia are commonly known as cell’s antenna. Cilia defects can result in a wide range of diseases known as ciliopathies, such as polydactyly, hydrocephalus, obesity and Marden-Walker syndrome [47]. Combined with previous researches [28, 29, 48, 49], we conclude that different genes in different species control the same phenotype through the regulation of ciliogenesis. This finding also emphasized the important role of cilia in limb development.

*ABCC4,* a member of the ATP-binding cassette transporter family is also known as multidrug resistance-associated protein 4 (*MRP4*) [50]. Most importantly, *ABCC4* encodes an important transmembrane transporter known to transport PGE2 and other molecules across cellular membranes. Further, it is also involved in ciliogenesis by mediating PGE2 signaling acts through a ciliary G-protein-coupled receptor, EP4, to upregulate cAMP synthesis and increase anterograde IFT [51–53]. As an important transmembrane protein, the mutation or mis-regulation of *ABCC4* results in the disorder of PGE2 transmembrane transport and further results in the misfunction of the stimulation of anterograde IFT through EP4 and protein kinase A (PKA). Jin et al. [51] reported that the T804M mutation in *ABCC4* showed cilium loss and cilium-associated phenotypes in zebrafish, including ventrally curved body axis, hydrocephalus, abnormal otolith number and laterality defects of the brain and other organs.

In our study, based on two uncorrelated families’ association analysis showed that some SNPs and INDELs in *ABCC4* were highly associated with PPD phenotype. Derived allele frequency analyses around *ABCC4* revealed that there were significant differences between affected and normal groups. Furthermore, a homozygous missense mutation was identified in all affected individuals but not in normal groups. More interestingly, we did not detect this mutation in large unpublished samples. Based on the important role of cilia in SHH single transduction and the function of *ABCC4* in cell ciliogenesis, we firmly suggest *ABCC4* as a new candidate gene for polydactyly in pigs. As the sample size in this study is relatively small and there are no additional expression data to further validate our results, we therefore suggest additional experimental verification in future studies.

## Conclusions

In this study, we identified *ABCC4* as a new candidate gene involved in PPD regulation possibly through ciliogenesis process. Our analysis detected a highly associated missense mutation in all affected individuals but not in normal groups. Prediction of protein structure and function with different methods showed that the mutated residue is located in an important domain for binding of other molecules. Mutation of the residue might have disturbed this function, resulting in the inactivation of *ABCC4* and further into the disorder of ciliogenesis.

To the best of our knowledge, this study is the first to report on the genetic variation identification of PPD in artiodactyls, and these results expand our understanding of PPD in further studies of limb malformation and enrich our knowledge on cilium as an important signaling center during vertebrate development. Finally, this study serves an example to study human diseases through the whole genome sequencing of pig as an animal model.

## Methods

### Samples

Three sibling Large White pigs from one litter were detected having preaxial polydactyly in a farm in Hebei province of China (Fig. 1), and all the available samples of this family were collected form the farm, including three PPD affected (F1-a1, F1-a2 and F1-a3) and two normal individuals (F0-1and F1-a4) from one litter, 11 normal individuals (F1-b1 to b11) from another litter. The remaining individuals (F0–2, F0–3 and F1-a5 to a9) were recorded but without tissue samples. Meanwhile, another Large White female case of PPD (F0–4) was identified in another farm in Yunnan province, China. Both the parents of this affected individual are normal, but there was no further information on this individual’s offspring or sibling. Two affected pigs (F1-a1 and F1-a3) were euthanized with pentobarbital sodium solution (100 mg/kg) to get the hooves for radiograph analysis.

### DNA extraction and sequencing

Ear tissue samples were collected from all the 17 available individuals and stored at − 80 °C. For each sample, 30 mg ear tissue was used to extract genomic DNA with the standard phenol-chloroform method. Quality and quantity were assessed by Nanodrop spectrophotometer 2000 and gel electrophoresis experiment. Library construction for re-sequencing was performed according to the Illumina library prepping protocols (Illumina Inc., San Diego, CA, USA) with the insert size of 380 bp. Pair-end (PE) reads length of 150 bp were generated from the resequencing libraries on the Illumina Hiseq X Ten platform (Illumina Inc., San Diego, CA, USA), and all individuals were re-sequenced above 30× depth of coverage.

### Quality control and mapping

Clean reads were trimmed from raw reads that were pre-processed to remove index adaptors and low-quality reads. Quality control for removing the low-quality reads was done based on the following criteria: Up to 10% of the read bases include “N” content in each sequenced read, up to 50% of the read bases include low quality (Q < = 5) bases content in any sequenced reads. After trimming, clean reads of each sample were aligned to the pig reference genome *Sus scrofa* 10.2 [54] using BWA program ver.0.7.10-r789 [55] with default parameters. SAMtools software [56] were used to convert the SAM files from different libraries belonging to the same individual to BAM files and sort and merge them. And then, PCR duplicated reads were marked based on Picard ver. 2.12.1 tools (http://broadinstitute.github.io/picard/). Finally, read depth and coverage of each individual were estimated based on the results of SAMtools ver. 1.9 [56].

### Variants identification and filtration

RealignerTargetCreator, IndelRealigner, and BaseRecalibrator tools in the Genome Analysis Toolkit (GATK) (ver. 3.7–0-gcfedb67) [57] were used for local realignment and base quality recalibration. SNPs were called using UnifiedGenotyper (default parameters) and filtered SNPs and small size INDELs were identified using the UnifiedGenotyper algorithm of GATK [57]. In order to reduce the error rate of calling variations, SNPs were filtered by VariantFiltration tools (QUAL≤40.0 || QD ≤ 2.0 || MQ ≤ 40.0 || FS ≥ 60.0 || MQRankSum ≤ − 12.5 || ReadPosRankSum< − 8.0 || MQ0 > = 4 & ((MQ0/(1.0*DP)) > 0.1) || -cluster 3 -window 10). Meanwhile, INDELs were filtered with the threshold of “QD < 2.0 || FS > 200.0 || ReadPosRankSum< -20.0” which recommended by GATK.

### Annotation and genotype phasing

All called variants were annotated with GTF file downloaded from the Ensembl website (ftp://ftp.ensembl.org/pub) with a custom Perl script. According to the genomic position, SNPs were classified into protein coding (synonymous or nonsynonymous) regions, introns, UTR and IGR. Moreover, to further annotate the SNPs in putative regulatory regions, we downloaded human genome annotation data from the ENCODE (Encyclopedia of DNA elements) [58] project and identified putative regulatory sequences in pig genome orthologous to human counterpart with an ENCODE annotation, such as transcription factor DNA-binding motif, transcription factor binding site and histone binding site. The annotation method was referred to Lü et al. [59]. Haplotype phasing was implemented with Beagle 4.1 [60].

### Reported candidate genes analysis

Based on the identified SNPs and INDELs in this study, the candidate loci which reported previously were graphically showed with Integrative Genomics Viewer (IGV) [40] to scan if there is any variation that was only occurred in affected individuals.

### Genome-wide screening of candidate variants

XP-EHH [34] scores were calculated with a local script to detect alleles frequencies fixed or nearly fixed and to compare the affected and normal groups. Moreover, F_ST_ [35] was estimated to provide insights into the genetic differentiation between the affected and normal groups based on whole-genome SNPs and INDELs with 10 kb window and 2 kb overlapping slides using VCFtools 0.1.14 [61]. In addition, F_ST_ of the three candidate genes (*ABCC4*, *LMBR1* and *SHH*) were calculated based on every SNP and INDEL loci.

### Case-control association studies

For analyzing of SNP effects related to the deformity of this two families, four affected and 13 available normal pigs from two families were compared using the basic case-control association analysis and family-based association test of PLINK v1.07 [36]. Meanwhile, based on the identified highly associated variations, genotyping and derived allele frequency were performed. Besides, we performed the family-based association test on all 16 individuals from the first family (the fourth affected individual F0–4 was excluded) to repeat the analyses for further support our results.

### Structure and function prediction

In order to further confirm the amino acid substitution (I85T) impact on protein structure and function, we used I-Mutant2.0 [41] (http://gpcr.biocomp.unibo.it/cgi/predictors/I-Mutant2.0/I-Mutant2.0.cgi) to predict the protein stability changes upon the mutation of rs791053563, the parameter of temperature was set to 38°Cand pH was 7.0. In addition, we used PROVERAN web server [42] (http://provean.jcvi.org/index.php) and HOPE [43] (http://www.cmbi.ru.nl/hope/) to predict this amino acid substitution’s impact on the biological function and structural effect of the protein. PROVERAN [42] will give a score of the variant and the default threshold is − 2.5, that is: variants with a score equal to or below − 2.5 are considered “deleterious” and variants with a score above − 2.5 are considered “neutral.” HOPE [43] is a web service that will produce a point mutation report based on the available information that collected and combined from a series of web services and databases. Moreover, in order to further compare the changes in protein structure caused by the missense mutation, we constructed the 3D structure of protein encoded by *ABCC4* with STRUM [44] (https://zhanglab.ccmb.med.umich.edu/STRUM/). All the prediction was based on the protein sequence of UniPortKB (ID: A0A287A6F6_PIG) or Ensembl (Transcript ID: ENSSSCT00000037963.1).

## Supplementary information

**Additional file 1: Table S1.** Information of all samples and their genome resequencing data characteristics.

**Additional file 2: Table S2.** Distribution and annotation of SNPs identified in this study. ENCODE indicate that the SNPs which were located in the homologous sequences of human counterparts have ENCODE annotations; Motif indicates that the SNPs which were located in the homologous sequences of transcription factor DNA-binding motifs of human counterparts; UTR, untranslated regions; CDS, coding sequence.

**Additional file 3: Table S3.** The highly remarkable regions (XP-EHH > 2) with annotation.

**Additional file 4: Table S4.** The highly differentiated windows based on whole-genome SNPs. Chr: Chromosome; Win_Start: Window’s Start; Win_End: Window’s End; N_SNPs: Number of SNPs.

**Additional file 5: Table S5.** The highly differentiated SNPs within the highly differentiated windows. The bold italic highlighted rows (*) represent the SNPs which identified in association test and for DAF analysis.

**Additional file 6: Figure S1.** The distribution of the highly differentiated SNPs.

**Additional file 7: TableS6.** The highly differentiated regions based on INDELs between PPD affected and normal pigs. Chr: Chromosome; Win_Start: Window’s Start; Win_End: Window’s End; N_SNPs: Number of INDELs.

**Additional file 8: Figure S2.** Plots of whole-genome screening of putatively loci which associated with PPD in the large family. (A) Genome wide SNPs plot of XP-EHH between the 3 affected (excluded F0–4) and 13 normal individuals. (B) F_ST_ plot of the whole selected SNPs between 3 affected and normal individuals. (C) F_ST_ plot between the 3 affected and normal individuals based on whole selected INDELs. (D) Manhattan plot of whole-genome association analysis based on whole SNPs of PPD phenotype.

**Additional file 9: Figure S3.** Schematic diagram of the relative position of *ABBC4* and *DZIP1*. Arrows indicate the direction of gene transcription.

**Additional file 10: Figure S4.** The F_ST_ comparison between three candidate genes based on all SNPs and INDELs. (A-C) F_ST_ plot based on all SNPs. (D-F) F_ST_ plot based on all INDELs.

**Additional file 11: Table S7.** Phased genotyping results of the top ten highly associated SNPs. The bold italic highlighted columns represent the affected individuals. The bold italic highlighted rows (*) represent the missense mutation SNP which located in the third exon of *ABCC4*.

**Additional file 12: Table S8.** The genotyping of the highly differentiated INDELs between PPD affected and normal pigs. Pos: Physical position; Ref: Reference allele; Alt: Altered allele; GT_A: Genotypes of cases; GT_U: Genotypes of controls; The number “0” represent reference allele and “1” represent altered allele; The number behind the genotype in brackets represent the number of this genotype’s individuals; DAF_A: Derived allele frequency in cases; DAF_U: Derived allele frequency in controls; IGR: intergenic region.

**Additional file 13: Table S9.** The allele frequency of the top ten highly associated SNPs between population in this study and unpublished data. Chr: Chromosome; Pos (Ssc10.2): Position in *Sus scrofa* 10.2; Ref: Reference allele; Ref_Freq_A: Reference allele frequency of population in this study; Ref_Freq_B: Reference allele frequency of population in unpublished data; Alt1: Alternative allele 1; Alt1_Freq_A: Alternative allele 1 frequency of population in this study; Alt1_Freq_B: Alternative allele 1 frequency of population in unpublished data; Alt2: Alternative allele 2; Alt2_Freq_A: Alternative allele 2 frequency of population in this study; Alt2_Freq_B: Alternative allele 2 frequency of population in unpublished data.

**Additional file 14: Table S10.** The genotype of the top ten highly associated SNPs between population in this study and the 559 unpublished data set. Pos: Physical position; Ssc: *Sus scrofa*; Ref: Reference allele; Alt: Altered allele; GT_A: Genotypes of affected cases; GT_U: Genotypes of unaffected controls; GT_C: Genotypes of validation data set; The number “0” represent reference allele and “1” represent altered allele; The number behind the genotype in brackets represent the number of this genotype’s individuals; IGR: Intergenic region. The bold italic highlighted rows (*) represent the SNPs which identified by genome scanning (Additional file 5: Table S5).

## Data Availability

All sequence data have been deposited in NCBI Sequence Read Achieve (SRA) with the Bioproject number PRJNA487539 or accessible through https://www.ncbi.nlm.nih.gov/bioproject/PRJNA487539. The experiment numbers for all the 17pigs are SRR7791346-SRR7791362. The prediction of protein function and structure was based on the protein sequence of UniPortKB (ID: A0A287A6F6_PIG) (https://www.uniprot.org/uniprot/A0A287A6F6) or Ensembl (Transcript ID: ENSSSCT00000037963.1) (http://asia.ensembl.org/*Sus_scrofa*/Transcript/Sequence_Protein?db=core;g=ENSSSCG00000026996;r=11:64086662-64309389;t=ENSSSCT00000037963).

## Abbreviations

PPD: Preaxial polydactyly
*ABCC4*: ATP-binding cassette sub-family C member 4;
SHH: Sonic Hedgehog
ZPA: Zone of Polarizing Activity
ZRS: Zone of Polarizing Activity Regulatory Sequence
IFT: Intraflagellar transport
UTR: Untranslated regions
CDS: Coding sequence
SSC: *Sus scrofa* chromosome
SNP: Single nucleotide polymorphism
INDEL: Insertion/Deletion
PGE2: Prostaglandin E2
IGR: Intergenic regions
DAF: Derived allele frequency
XP-EHH: Cross Population Extended Haplotype Homozygosity
CNV: Copy number variations
SV: Structural variants
*CYLD*: Cylindromatosis
F_ST_: Fixation index

## References

[1] Faust KC, Kimbrough T, Oakes JE, Edmunds JO, Faust DC. Polydactyly of the hand. Am J Orthop (Belle Mead, NJ). 2015;44(5):E127–34. 25950541

[2] Malik S (2014). Polydactyly: phenotypes, genetics and classification. Clin Genet.

[3] Wassel HD (1969). The results of surgery for polydactyly of the thumb. A review. Clin Orthop Relat Res.

[4] Deng H, Tan T. Advances in the Molecular Genetics of Non-syndromic Syndactyly. Curr Genomics. 2015;16(3):183–93. 10.2174/1389202916666150317233103 PMC4460222 26069458

[5] Guo B, Lee SK, Paksima N (2013). Polydactyly: a review. Bull Hosp Joint Dis (2013).

[6] Farrugia MC, Calleja-Agius J (2016). Polydactyly: a review. Neonatal Network.

[7] Riddle RD, Johnson RL, Laufer E, Tabin C (1993). Sonic hedgehog mediates the polarizing activity of the ZPA. Cell.

[8] Torok MA, Gardiner DM, Izpisúa-Belmonte JC, Bryant SV (1999). Sonic hedgehog (shh) expression in developing and regenerating axolotl limbs. J Exp Zool A Ecol Genet Physiol.

[9] Lettice LA, Hill RE (2005). Preaxial polydactyly: a model for defective long-range regulation in congenital abnormalities. Curr Opin Genet Dev.

[10] McMahon AP, Ingham PW, Tabin CJ (2003). 1 developmental roles and clinical significance of hedgehog signaling. Curr Top Dev Biol.

[11] Lettice LA, Horikoshi T, Heaney SJ, van Baren MJ, van der Linde HC, Breedveld GJ, Joosse M, Akarsu N, Oostra BA, Endo N (2002). Disruption of a long-range cis-acting regulator for Shh causes preaxial polydactyly. Proc Natl Acad Sci U S A.

[12] Lettice LA (2003). A long-range Shh enhancer regulates expression in the developing limb and fin and is associated with preaxial polydactyly. Hum Mol Genet.

[13] Lettice LA, Hill AE, Devenney PS, Hill RE (2008). Point mutations in a distant sonic hedgehog cis-regulator generate a variable regulatory output responsible for preaxial polydactyly. Hum Mol Genet.

[14] Lettice LA, Williamson I, Devenney PS, Kilanowski F, Dorin J, Hill RE (2014). Development of five digits is controlled by a bipartite long-range cis-regulator. Development.

[15] Anderson E, Peluso S, Lettice LA, Hill RE (2012). Human limb abnormalities caused by disruption of hedgehog signaling. Trends Genet.

[16] Zhang Z, Sui P, Dong A, Hassell J, Cserjesi P, Chen YT, Behringer RR, Sun X (2010). Preaxial polydactyly: interactions among ETV, TWIST1 and HAND2 control anterior-posterior patterning of the limb. Development.

[17] te Welscher P, Fernandez-Teran M, Ros MA, Zeller R (2002). Mutual genetic antagonism involving GLI3 and dHAND prepatterns the vertebrate limb bud mesenchyme prior to SHH signaling. Genes Dev.

[18] Wang B, Fallon JF, Beachy PA (2000). Hedgehog-regulated processing of Gli3 produces an anterior/posterior repressor gradient in the developing vertebrate limb. Cell.

[19] Litingtung Y, Dahn RD, Li Y, Fallon JF, Chiang C (2002). Shh and Gli3 are dispensable for limb skeleton formation but regulate digit number and identity. Nature.

[20] Quinn ME, Haaning A, Ware SM (2012). Preaxial polydactyly caused by Gli3 haploinsufficiency is rescued by Zic3 loss of function in mice. Hum Mol Genet.

[21] Sheth R, Bastida MF, Ros M (2007). Hoxd and Gli3 interactions modulate digit number in the amniote limb. Dev Biol.

[22] Wilson CW, Stainier DYR (2010). Vertebrate Hedgehog signaling: cilia rule. BMC Biol.

[23] Goetz SC, Anderson KV (2010). The primary cilium: a signalling Centre during vertebrate development. Nat Rev Genet.

[24] Goetz SC, Ocbina PJ, Anderson KV (2009). The primary cilium as a hedgehog signal transduction machine. Methods Cell Biol.

[25] Huangfu D, Anderson KV (2006). Signaling from Smo to ci/Gli: conservation and divergence of hedgehog pathways from Drosophila to vertebrates. Development.

[26] Eggenschwiler JT, Anderson KV (2007). Cilia and developmental signaling. Annu Rev Cell Dev Biol.

[27] Merrill AE, Merriman B, Farrington-Rock C, Camacho N, Sebald ET, Funari VA, Schibler MJ, Firestein MH, Cohn ZA, Priore MA (2009). Ciliary abnormalities due to defects in the retrograde transport protein DYNC2H1 in short-rib polydactyly syndrome. Am J Hum Genet.

[28] Hu Y, Wu Q, Ma S, Ma T, Shan L, Wang X, Nie Y, Ning Z, Yan L, Xiu Y (2017). Comparative genomics reveals convergent evolution between the bamboo-eating giant and red pandas. Proc Natl Acad Sci.

[29] Yang Y, Ran J, Liu M, Li D, Li Y, Shi X, Meng D, Pan J, Ou G, Aneja R (2014). CYLD mediates ciliogenesis in multiple organs by deubiquitinating Cep70 and inactivating HDAC6. Cell Res.

[30] Lawrence PA, Casal J (1999). Struhl G: hedgehog and engrailed: pattern formation and polarity in the Drosophila abdomen. Development.

[31] Kondoh S, Sugawara H, Harada N, Matsumoto N, Ohashi H, Sato M, Kantaputra PN, Ogino T, Tomita H, Ohta T (2002). A novel gene is disrupted at a 14q13 breakpoint of t (2; 14) in a patient with mirror-image polydactyly of hands and feet. J Hum Genet.

[32] Firulli BA, Redick BA, Conway SJ, Firulli AB (2007). Mutations within helix I of Twist1 result in distinct limb defects and variation of DNA binding affinities. J Biol Chem.

[33] Klopocki E, Kähler C, Foulds N, Shah H, Joseph B, Vogel H, Lüttgen S, Bald R, Besoke R, Held K (2012). Deletions in PITX1 cause a spectrum of lower-limb malformations including mirror-image polydactyly. Eur J Hum Genet.

[34] Sabeti PC, Varilly P, Fry B, Lohmueller J, Hostetter E, Cotsapas C, Xie X, Byrne EH, McCarroll SA, Gaudet R (2007). Genome-wide detection and characterization of positive selection in human populations. Nature.

[35] Holsinger KE, Weir BS (2009). Genetics in geographically structured populations: defining, estimating and interpreting FST. Nat Rev Genet.

[36] Purcell S, Neale B, Todd-Brown K, Thomas L, Ferreira MA, Bender D, Maller J, Sklar P, De Bakker PI, Daly MJ (2007). PLINK: a tool set for whole-genome association and population-based linkage analyses. Am J Hum Genet.

[37] Sekimizu K, Nishioka N, Sasaki H, Takeda H, Karlstrom RO, Kawakami A (2004). The zebrafish iguana locus encodes Dzip1, a novel zinc-finger protein required for proper regulation of hedgehog signaling. Development.

[38] Wolff C, Roy S, Lewis KE, Schauerte H, Joerg-Rauch G, Kirn A, Weiler C, Geisler R, Haffter P (2004). Ingham PW: iguana encodes a novel zinc-finger protein with coiled-coil domains essential for hedgehog signal transduction in the zebrafish embryo. Genes Dev.

[39] Glazer AM, Wilkinson AW, Backer CB, Lapan SW, Gutzman JH, Cheeseman IM, Reddien PW (2010). The Zn finger protein Iguana impacts hedgehog signaling by promoting ciliogenesis. Dev Biol.

[40] Thorvaldsdóttir H, Robinson JT, Mesirov JP (2013). Integrative genomics viewer (IGV): high-performance genomics data visualization and exploration. Brief Bioinform.

[41] Capriotti E, Fariselli P, Casadio R (2005). I-Mutant2.0: predicting stability changes upon mutation from the protein sequence or structure. Nucleic Acids Res.

[42] Choi Y, Chan AP (2015). PROVEAN web server: a tool to predict the functional effect of amino acid substitutions and indels. Bioinformatics.

[43] Venselaar H, te Beek TAH, Kuipers RKP, Hekkelman ML, Vriend G (2010). Protein structure analysis of mutations causing inheritable diseases. An e-Science approach with life scientist friendly interfaces. BMC Bioinformatics.

[44] Quan L, Lv Q, Zhang Y (2016). STRUM: structure-based prediction of protein stability changes upon single-point mutation. Bioinformatics.

[45] Gieling ET, Schuurman T, Nordquist RE, van der Staay FJ, Hagan JJ (2011). The pig as a model animal for studying cognition and neurobehavioral disorders. Molecular and functional models in neuropsychiatry.

[46] Murdoch JN, Copp AJ (2010). The relationship between sonic hedgehog signaling, cilia, and neural tube defects. Birth Defects Res A Clin Mol Teratol.

[47] Badano JL, Mitsuma N, Beales PL, Katsanis N (2006). The ciliopathies: an emerging class of human genetic disorders. Annu Rev Genomics Hum Genet.

[48] Hao L, Scholey JM (2009). Intraflagellar transport at a glance. J Cell Sci.

[49] Haycraft CJ, Banizs B, Aydin-Son Y, Zhang Q, Michaud EJ, Yoder BK (2005). Gli2 and Gli3 localize to cilia and require the intraflagellar transport protein polaris for processing and function. PLoS Genet.

[50] Lee K, Belinsky MG, Bell DW, Testa JR, Kruh GD (1998). Isolation of MOAT-B, a widely expressed multidrug resistance-associated protein/canalicular multispecific organic anion transporter-related transporter. Cancer Res.

[51] Jin D, Ni TT, Sun J, Wan H, Amack JD, Yu G, Fleming J, Chiang C, Li W, Papierniak A (2014). Prostaglandin signaling regulates ciliogenesis by modulating intraflagellar transport. Nat Cell Biol.

[52] Barbry P, Zaragosi L-E (2014). An ABC of ciliogenesis. Nat Cell Biol.

[53] Abla N, Chinn LW, Nakamura T, Liu L, Huang CC, Johns SJ, Kawamoto M, Stryke D, Taylor TR, Ferrin TE (2008). The human multidrug resistance protein 4 (MRP4, ABCC4): functional analysis of a highly polymorphic gene. J Pharmacol Exp Ther.

[54] Groenen MA, Archibald AL, Uenishi H, Tuggle CK, Takeuchi Y, Rothschild MF, Rogel-Gaillard C, Park C, Milan D, Megens H-J (2012). Analyses of pig genomes provide insight into porcine demography and evolution. Nature.

[55] Li H, Durbin R (2009). Fast and accurate short read alignment with burrows–wheeler transform. Bioinformatics.

[56] Li H, Handsaker B, Wysoker A, Fennell T, Ruan J, Homer N, Marth G, Abecasis G, Durbin R (2009). The sequence alignment/map format and SAMtools. Bioinformatics.

[57] McKenna A, Hanna M, Banks E, Sivachenko A, Cibulskis K, Kernytsky A, Garimella K, Altshuler D, Gabriel S, Daly M (2010). The genome analysis toolkit: a MapReduce framework for analyzing next-generation DNA sequencing data. Genome Res.

[58] The ENCODE (2004). (ENCyclopedia of DNA elements) project. Science.

[59] Lü MD, Han XM, Ma YF, Irwin DM, Yun G, Deng JK, Adeola AC, Xie HB, Zhang YP: Genetic variations associated with six-white-point coat pigmentation in Diannan small-ear pigs. Scientific reports 2016, 6:27534. 10.1038/srep27534 PMC4897638 27270507

[60] Browning BL, Browning SR (2016). Genotype imputation with millions of reference samples. Am J Hum Genet.

[61] Danecek P, Auton A, Abecasis G, Albers CA, Banks E, DePristo MA, Handsaker RE, Lunter G, Marth GT, Sherry ST. The variant call format and VCFtools. Bioinformatics. 2011;27(15):2156–8. 10.1093/bioinformatics/btr330 PMC3137218 21653522

