# Supplementary figures and images for: Whole genome resequencing reveals an association of ABCC4 variants with preaxial polydactyly in pigs
Source: BMC Genomics. 2020 Mar 30;21:268. doi: 10.1186/s12864-020-6690-1 (PMC7106734; doi:10.1186/s12864-020-6690-1)

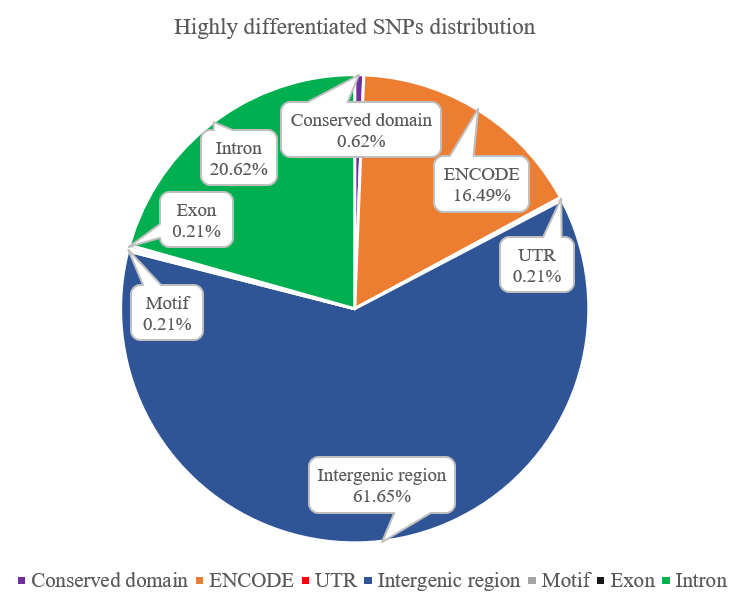

Supplement: Supplementary file 6 — Additional file 6: Figure S1. The distribution of the highly differentiated SNPs. [file 12864_2020_6690_MOESM6_ESM.tif]

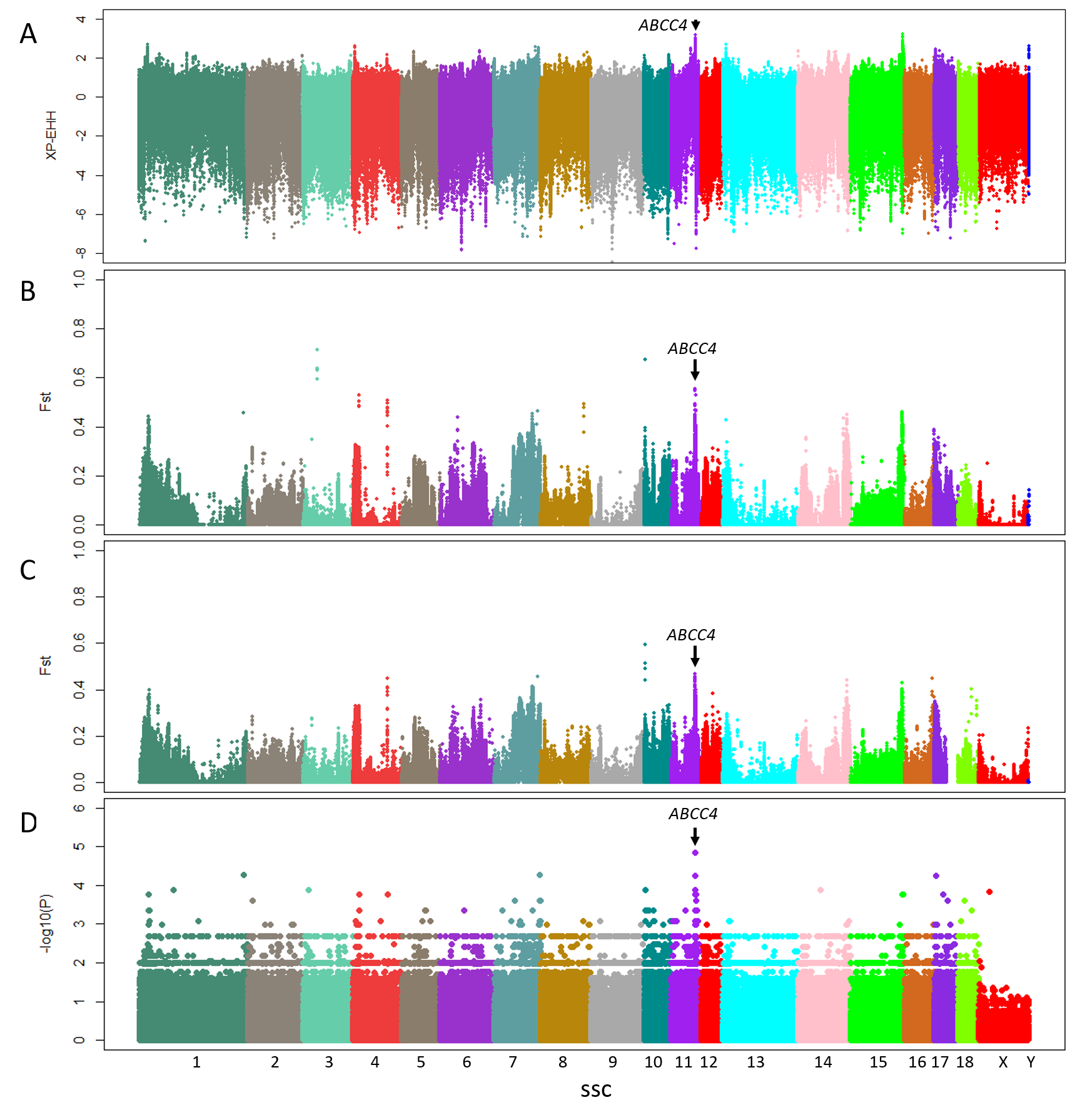

Supplement: Supplementary file 8 — Additional file 8: Figure S2. Plots of whole-genome screening of putatively loci which associated with PPD in the large family. (A) Genome wide SNPs plot of XP-EHH between the 3 affected (excluded F0–4) and 13 normal individuals. (B) FST plot of the whole selected SNPs between 3 affected and normal individuals. (C) FST plot between the 3 affected and normal individuals based on whole selected INDELs. (D) Manhattan plot of whole-genome association analysis based on whole SNPs of PPD phenotype. [file 12864_2020_6690_MOESM8_ESM.tif]

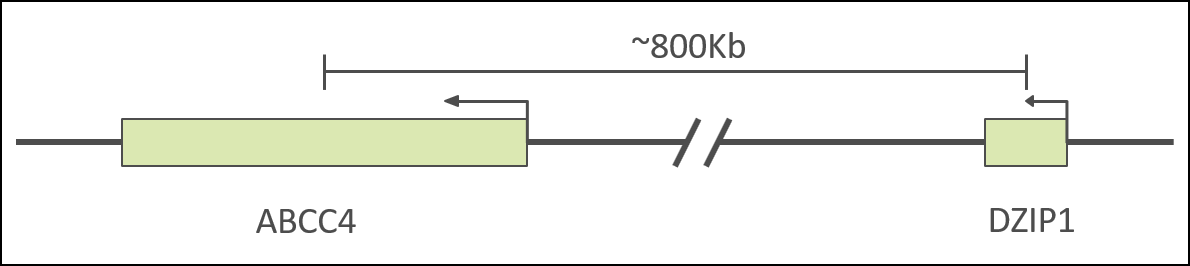

Supplement: Supplementary file 9 — Additional file 9: Figure S3. Schematic diagram of the relative position of ABBC4 and DZIP1. Arrows indicate the direction of gene transcription. [file 12864_2020_6690_MOESM9_ESM.tif]

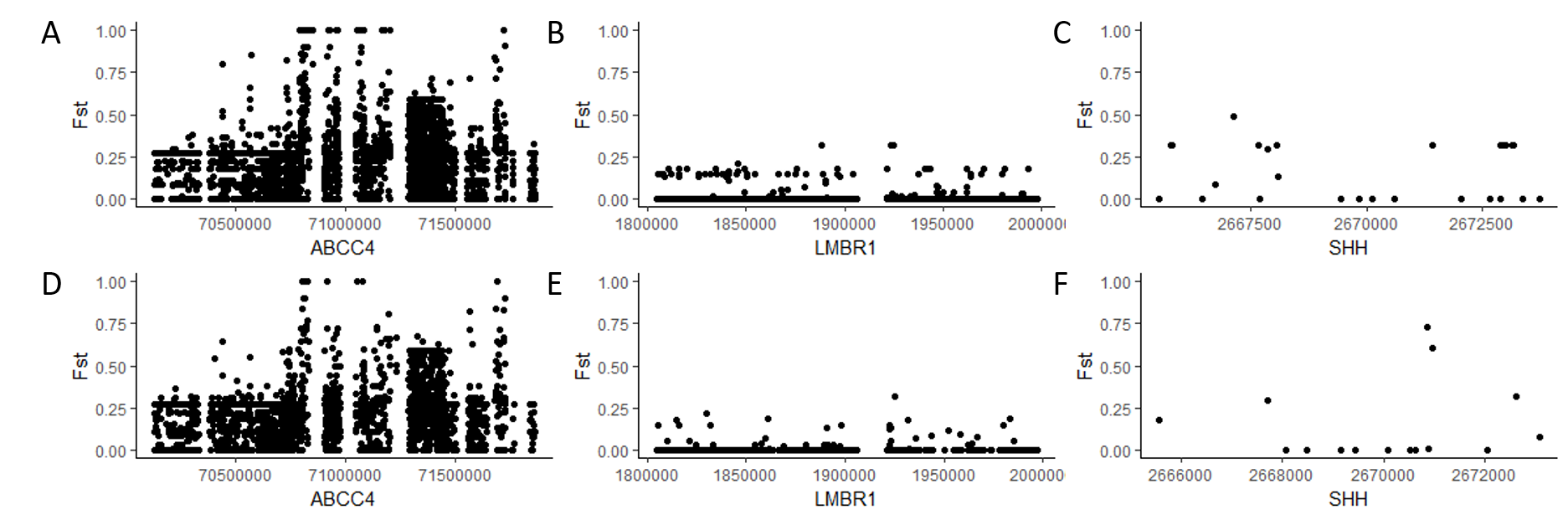

Supplement: Supplementary file 10 — Additional file 10: Figure S4. The FST comparison between three candidate genes based on all SNPs and INDELs. (A-C) FST plot based on all SNPs. (D-F) FST plot based on all INDELs. [file 12864_2020_6690_MOESM10_ESM.tif]
